# Allogeneic adipose MSCs and autologous PRP for chronic non-healing wound in a cat

**DOI:** 10.17221/93/2024-VETMED

**Published:** 2025-04-28

**Authors:** Natthima Suwan, Sirinya Jenjittikul, Rattichid Tiratrakoonseree, Chanyapat Jiradanaipat, Sasipat Teerawongsuwan, Wipawadee Phathomrapeepong, Warangkhana Phanwanich, Ruttachuk Rungsiwiwut

**Affiliations:** ^1^Faculty of Medicine, Srinakharinwirot University, Nakhon Nayok, Thailand; ^2^Department of Anatomy, Faculty of Medicine, Srinakharinwirot University, Bangkok, Thailand; ^3^Wipawadee animal clinic, Bangkok, Thailand; ^4^Department of Biomedical Engineering, Faculty of Engineer, Mahidol University, Nakhon Pathom, Thailand; ^5^Petaneer Co., Ltd. Salaya, Nakhon Pathom, Thailand

**Keywords:** bite wound, regenerative veterinary medicine, stem cells

## Abstract

Management of chronic non-healing wounds in cats requires a comprehensive approach. This report describes the treatment of a severe open skin wound on the skull using a combined approach involving allogeneic adipose-derived mesenchymal stem cells (MSCs) and autologous platelet-rich plasma (PRP). A 12-year-old neutered male mixed-breed domestic cat presented with a non-healing chronic wound on the skull. The wound extended from the orbital to the occipital area and from the left to the right temporal region. Laboratory test results were positive for feline immunodeficiency virus and impaired kidney function. Sensitivity tests revealed resistance to several antibiotics. Due to limited skin reconstruction options, MSCs were administered subcutaneously at the wound edge once a month for three months. PRP was collected one month after the initial MSC administration and injected at the wound edge monthly between MSC treatments. The wound diameter was measured daily during saline cleaning. The cat received protein-supplemented food daily. Wound healing was observed two weeks after the MSC administration, gradually decreasing in size and closing completely within 5 months. This case demonstrates the successful application of MSCs and PRP for treating chronic wounds in cats.

Chronic wounds in felines pose substantial clinical challenges due to their multifactorial aetiologies. Persistent infections, trauma, foreign bodies, neoplasia, immune-mediated diseases, metabolic disorders (e.g., diabetes mellitus), and nutritional deficiencies can disrupt wound healing, resulting in prolonged inflammation and disrupted tissue regeneration (Balsa and Culp 2015). Complications from chronic wounds greatly diminish the quality of life, often leading to secondary infections with antibiotic-resistant bacteria, such as *Staphylococcus* and *Pseudomonas* species (Malik et al. 2005), which can cause abscesses, excessive scarring, and fibrosis, all contributing to functional impairment. Chronic inflammation and persistent pain can cause systemic illnesses and behavioural abnormalities in cats.

The effective treatment of chronic wounds requires a comprehensive multimodal strategy. Initial disinfection with sterile saline or an antiseptic solution is required (Fernandez et al. 2022). Surgical, enzymatic, or autolytic debridement is essential for removing necrotic tissue and promoting healthy tissue growth. Depending on the culture and sensitivity, systemic antibiotics and topical antimicrobials, such as silver sulfadiazine, may also be applied to control infection (Drudi et al. 2018). Maintaining a moist wound environment with proper dressings (e.g., hydrocolloid or foam) is imperative to ensure optimal healing. Nutritional support is also crucial; a diet rich in essential vitamins and minerals, together with pain relief by veterinary professionals via NSAIDs or opioids, enhances recovery (Taylor et al. 2024). Adjunctive therapies such as low-level laser therapy, hyperbaric oxygen therapy, and growth factors, such as platelet-rich plasma, have all been shown to improve healing outcomes (Gouveia et al. 2021; Changrani-Rastogi et al. 2023). Surgical intervention may be required in extreme cases. Consistent follow-up is necessary to monitor progress and adjust treatment accordingly.

Mesenchymal stem cells (MSCs) are emerging as effective treatments for chronic wounds in cats. MSCs promote angiogenesis, control inflammation, and differentiate into distinct cell types essential for tissue repair via paracrine signalling. Studies have shown that the MSCs decrease scarring, enhance wound quality, and accelerate wound closure. In addition, their immunomodulatory properties help reduce chronic inflammation (Park et al. 2021). The MSCs provide a new therapeutic option for managing feline wounds, as they can be injected locally or integrated into biocompatible scaffolds. Platelet-rich plasma (PRP) is also gaining popularity because of its wound-healing benefits (Changrani-Rastogi et al. 2023). Concentrated in cat plasma, the PRP is rich in growth factors essential for tissue repair, promoting angiogenesis, and accelerating epithelialisation (Amable et al. 2013). Such autologous treatment is associated with a lower risk of immunogenic reactions, making it a safe therapeutic alternative.

This case highlights innovative treatments using the allogeneic adipose-derived (ADMSCs) and autologous PRP to manage a severe chronic wound in a cat.

## Case description

**Figure 1:**
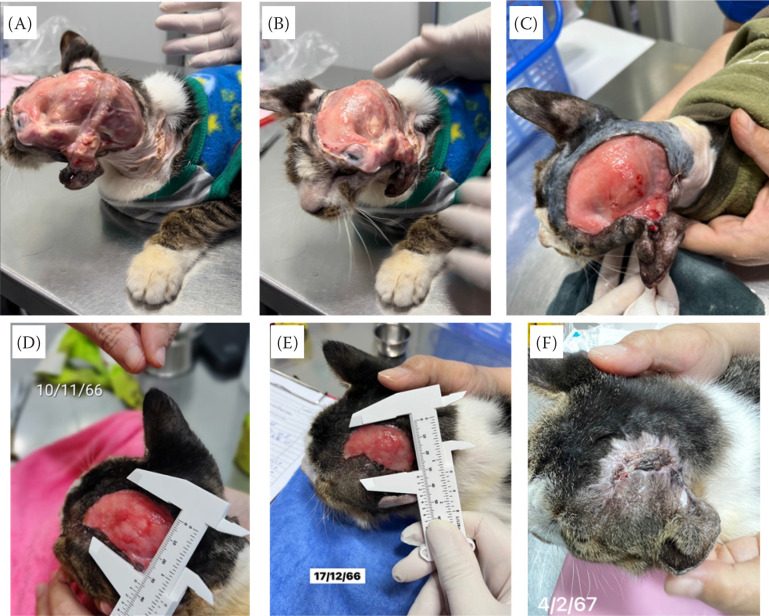
Photographs of chronic wounds healing of a 12-year-old neutered male mixed-breed domestic cat (A,B) The cat presented with bite wounds that had progressed into chronic, non-healing lesions, extending from the orbital to the occipital regions and spanning across the left and right temporal areas. The affected wound area measured over 10 cm². (C) Two weeks after the administration of mesenchymal stem cells (MSCs), the wound and overall health condition showed marked improvement. (D) Two months after MSC and platelet-rich plasma (PRP) administration. (E) Three months after MSC and PRP administration, the wound area had reduced to less than half of its original size. (F) Four months post-administration, the wound had closed and was covered by a scab

**Figure 2:**
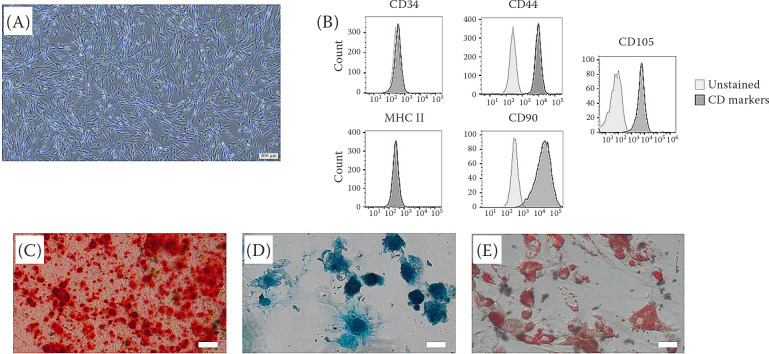
Morphology and characterisation of feline MSCs Feline MSCs were derived from the adipose tissue of cats that had undergone ovariohysterectomy. The isolated cells exhibited key characteristics of MSCs, including adherence to plastic surfaces and fibroblast-like morphology (A), high expression of surface markers CD44, CD90, and CD105, and lack of expression of CD34 and MHC II (B). The cells could differentiate into (C) osteoblast stained with Alizarin red, (D) chondrocytes stained with Alcian blue, and (E) adipocytes stained with Oil Red O. Scale bars = 200 μm CD = cluster of differentiation; MHC = major histocompatibility complex; MSCs = mesenchymal stem cells

A 12-year-old neutered male mixed-breed domestic cat presented with a non-healing chronic wound on the skull (Figure 1A,B). The cat had suffered from this wound for over two months before being referred to the Wipawadee Animal Clinic in Bangkok. The cat had sustained bite wounds that developed into non-healing wounds, extending from the orbital to the occipital area, spanning the left to right temporal regions. The wound area was larger than 10 cm². Previous interventions included laser wound therapy and surgical wound closure, both of which were unsuccessful. Physical examination revealed hypothermia (36.8 °C), with normal lung sounds and a body condition score indicative of cachexia. Additionally, the capillary refill time was 3 s and the mucous membranes appeared pale pink with slight dryness. The rapid detection of feline leukaemia virus and feline immunodeficiency virus (FIV) using whole blood was positive for FIV. The cat’s kidney function was assessed through a complete blood count, serum biochemical analysis, and urinalysis, revealing impaired kidney function. Antibiotic sensitivity testing, performed using swabs of wound exudate followed by bacterial culture, demonstrated resistance to multiple antibiotics, including amoxicillin, amikacin, cephalexin, doxycycline, and enrofloxacin. Due to the limitations of surgical skin reconstruction and the high risk of anaesthesia, the owner consented to our treatment plan involving local allogeneic MSC injections in combination with wound dressings and nutritional supplementation. Feline allogeneic ADMSCs were further isolated, cultured, and characterised as part of other research projects. Protocols for deriving the feline MSCs were approved by the Institutional Review Board of Srinakharinwirot University (COA/AE-017-2563). Feline allogeneic ADMSCs exhibited typical morphological and molecular features as shown in Figure 2. Allogeneic ADMSCs were further tested before treatment to ensure they were free of endotoxins and Mycoplasma contamination.

During the first three weeks, the cat underwent daily wound cleaning using only saline solution, and the wound was covered with saline-soaked gauze before bandaging. No antibiotic cream or injection was applied during this period. Commercial food to support kidney function (Royal Canin^®^), food supplements to reduce inflammation (Antinol^®^), and five Thai edible plants extract to boost the immune system (Canvirol^®^ 170 mg/capsule) were provided twice a day. In the third week, although the wound area had not decreased substantially, its condition improved, as evidenced by a change in colour from pale to light pink. Additionally, the cat’s body weight increased from 3.0 kg to 3.6 kg, representing a 20% increase. Before daily cleaning and bandaging, a total of 1 × 10^7^ allogeneic MSCs in 0.5 ml of saline solution was prepared for subcutaneous injection. The injection was administered approximately 0.5 cm away from the edge of the wound, with 0.1 ml of cell suspension injected at each site around the wound. The MSC injection was administered to the cat while fully conscious and without anaesthesia. The cat showed no signs of allergic reactions and no notable negative signs 24 h after the MSC injection. After two weeks, the wound and the cat’s overall health progressively improved (Figure 1C).

**Figure 3:**
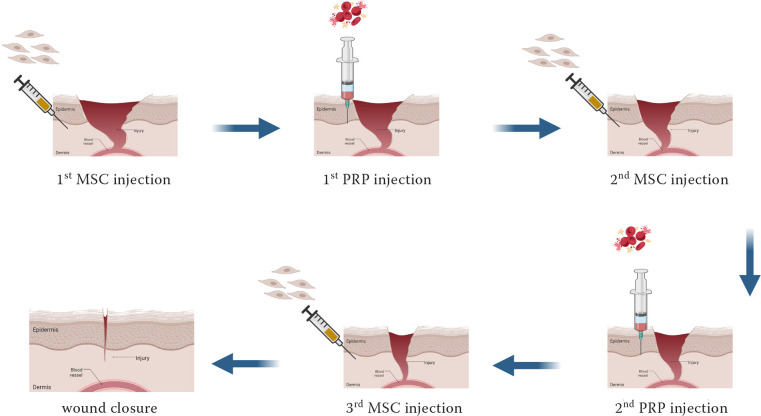
Timeline of allogeneic adipose-derived mesenchymal stem cells (ADMSCs) and allogeneic platelet-rich plasma (PRP) administration The schematic timeline illustrates the administration of allogeneic MSCs and allogeneic PRP administration. Allogeneic ADMSCs were administered subcutaneously at the wound edge once a month for three months. Autologous PRP was collected one month after the initial MSC administration and injected at the wound edge monthly between MSC treatments (created with BioRender.com)

After discussing with the owner and receiving consent to apply autologous PRP to stimulate wound healing, 5 ml of whole blood was collected via jugular venipuncture into a heparin-coated collection tube. According to a previous report (Angelou et al. 2022), autologous PRP was prepared at the Stem Cell Laboratory, Faculty of Medicine, Srinakharinwirot University. A total of 0.5 ml of autologous PRP, diluted in autologous serum, was administered around the wound area like that for allogeneic ADMSCs. The timeline for MSC and PRP administration is shown in Figure 3.

Three MSC and two PRP injections combined with saline solution wound cleaning, dressing, and supplementation with high-quality food and supplements resulted in complete healing of the chronic wound within five months without the need for surgical skin reconstruction (Figure 1D–F). After three months of follow-up, the owner confirmed that the cat had experienced no complications in the wound area and had a good quality of life.

## DISCUSSION AND CONCLUSION

Wound healing is a complex skin repair process following injury, involving different cell types and the intricate actions of multiple factors. The method comprises four phases: haemostasis, inflammation, proliferation, and remodelling (Ohnstedt et al. 2019). In cats, wound healing complications can arise from various factors, such as poor management, poor nutrition, or secondary infections, leading to severe hypoxia and a complex inflammatory environment (Zhang et al. 2023).

In the present report, based on the wound conditions observed upon arrival at the referring animal clinic, as well as the medical history provided, the wounds had persisted for over two months, which complicated the evaluation of the wound healing process. Therefore, the regenerative wound management strategy aimed to reduce inflammation, accelerate wound healing, and facilitate tissue repair without surgical wound closure or antibiotic application. Wound cleaning with saline solution is a well-documented practice in veterinary medicine, including in cats. Saline (0.9% sodium chloride solution) is commonly used because it is isotonic, meaning that it has the same salt concentration as the body tissues, minimising irritation and promoting a more conducive environment for wound healing (Fernandez et al. 2022). Moist wound healing has emerged as a cornerstone in the management of chronic wounds, offering several advantages, including faster tissue repair, reduced pain, and minimised scarring. This approach promotes cell migration by maintaining a moist environment, supporting autolytic debridement, and preventing desiccation of the wound bed, all of which are essential for optimal healing. Various materials, such as hydrocolloids and hydrogels, foam dressings and alginates, and transparent film dressings, are available to facilitate moist wound healing, each tailored to the specific wound characteristics of different wound healing phases (Erwin et al. 2021; Niculescu and Grumezescu 2022).

Excessive inflammation is a key feature of chronic wound progression. MSCs decrease inflammation through their immunomodulatory effects, which are mediated by cell-cell interactions and paracrine growth factors. MSCs further modify the activities of T, B, and natural killer cells, activate regulatory T cells, and release immunosuppressive factors (Otero-Vinas and Falanga 2016). In addition to reducing wound inflammation, growth factors such as bFGF, VEGF, HGF, and IL-6 secreted by MSCs also play roles in angiogenesis, endothelialisation, remodelling, and promoting the survival and proliferation of targeted cells (Tao et al. 2016). The positive effect of MSC application on wound healing in cats has been previously reported (Kim et al. 2022). In addition to MSCs, autologous PRP was used in this study. Clinical studies and case reports have documented the successful use of PRP in various feline wounds, including surgical incisions, traumatic injuries, and chronic non-healing ulcers (Verma et al. 2023). PRP can be administered through local injections directly into the wound or topically applied to a biocompatible scaffold or dressing (Liu et al. 2022). The interaction between ADMSCs and PRP during wound healing leverages the strength of both components. PRP enhances the regenerative capacity of ADMSCs by providing a conducive environment rich in growth factors, while ADMSCs contribute to effective tissue repair via differentiation and paracrine effects. This combination therapy is promising for advancing veterinary and human medicine wound management.

In conclusion, this case report demonstrated the successful dual application of MSCs and PRP for treating chronic wounds in cats. Despite the encouraging outcomes, standardised protocols for preparing and applying MSCs and PRP in cats are lacking. Further studies are necessary to establish the optimal dose, application frequency, and long-term efficacy.
